# Uncovering the Effect and Mechanism of *Rhizoma Corydalis* on Myocardial Infarction Through an Integrated Network Pharmacology Approach and Experimental Verification

**DOI:** 10.3389/fphar.2022.927488

**Published:** 2022-07-22

**Authors:** Jingyan Li, Junxuan Wu, Junying Huang, Yuanyuan Cheng, Dawei Wang, Zhongqiu Liu

**Affiliations:** ^1^ Guangdong Key Laboratory for Translational Cancer Research of Chinese Medicine, Joint Laboratory for Translational Cancer Research of Chinese Medicine of the Ministry of Education of the People’s Republic of China, Guangdong-Hong Kong-Macau Joint Lab on Chinese Medicine and Immune Disease Research International, International Institute for Translational Chinese Medicine, School of Pharmaceutical Science, Guangzhou University of Chinese Medicine, Guangzhou, China; ^2^ Shunde Hospital of Guangzhou University of Translational Chinese Medicine, Foshan, China; ^3^ The Second Affiliated Hospital of Guangzhou University of Chinese Medicine, Guangdong Provincial Hospital of Chinese Medicine, Guangzhou, China; ^4^ College of Life Sciences, Guangzhou University, Guangzhou, China

**Keywords:** *Corydalis yanhusuo*, tetrahydropalmatine, myocardial infarction, network pharmacology, apoptosis, PI3k/Akt signaling pathway

## Abstract

**Background:** Myocardial infarction (MI), characterized by reduced blood flow to the heart, is a coronary artery disorder with the highest morbidity and mortality among cardiovascular diseases. Consequently, there is an urgent need to identify effective drugs to treat MI. *Rhizoma Corydalis* (RC) is the dry tuber of *Corydalis yanhusuo* W.T. Wang, and is extensively applied in treating MI clinically in China. Its underlying pharmacological mechanism remains unknown. This study aims to clarify the molecular mechanism of RC on MI by utilizing network pharmacology and experimental verification.

**Methods:** Based on network pharmacology, the potential targets of the RC ingredients and MI-related targets were collected from the databases. Furthermore, core targets of RC on MI were identified by the protein-protein interaction (PPI) network and analyzed with Gene Ontology (GO) analysis and the Kyoto Encyclopedia of Genes and Genomes (KEGG) pathway enrichment analysis. Molecular docking was used to validate the binding affinity between the core targets and the bioactive components. Oxygen-glucose deprivation (OGD) was performed on H9c2 cells to mimic MI *in vitro*. A Cell Counting Kit-8 assay was used to assess the cardioprotective effect of the active ingredient against OGD. Western blot analysis and RT-qPCR were used to measure the cell apoptosis and inflammation level of H9c2 cells.

**Results:** The network pharmacology obtained 60 bioactive components of RC, 431 potential targets, and 1131 MI-related targets. In total, 126 core targets were screened according to topological analysis. KEGG results showed that RC was closely related to the phosphatidylinositol 3-kinase (PI3K)/Protein kinase B (PKB, also called Akt) signaling pathway. The experimental validation data showed that tetrahydropalmatine (THP) pretreatment preserved cell viability after OGD exposure. THP suppressed cardiomyocyte apoptosis and inflammation induced by OGD, while LY294002 blocked the inhibition effect of THP on OGD-induced H9c2 cell injury. Moreover, the molecular docking results indicated that THP had the strongest binding affinity with Akt over berberine, coptisine, palmatine, and quercetin.

**Conclusion:** THP, the active ingredient of RC, can suppress OGD-induced H9c2 cell injury by activating the PI3K/Akt pathway, which in turn provides a scientific basis for a novel strategy for MI therapy and RC application.

## Introduction

Myocardial infarction (MI), characterized by myocardial necrosis, is a major cause of morbidity and mortality worldwide, resulting in an estimated 7.4 million deaths per year (Xu et al., 2020). The acknowledged pathophysiology of MI is that thrombus formation in a coronary artery induces a reduction in myocardial perfusion (Frangogiannis, 2015). The general therapy includes coronary artery bypass grafting (CABG), percutaneous coronary intervention (PCI), and pharmacological management (Anderson and Morrow, 2017). However, reperfusion therapies, such as CABG and PCI, cause myocardial ischemia/reperfusion injury (MI/RI); antiplatelet therapy increases bleeding risk; and statin therapy may lead to drug-induced hepatic injury (Meurer and Cohen, 2020; Zheleva-Kyuchukova and Gelev, 2020; He et al., 2022). Consequently, identification of effective drugs to treat MI is urgently required.

*R. Corydalis* (RC) is the dried tuber of *C. yanhusuo* W.T. Wang, which belongs to the Papaveraceae family. RC was first recorded by Shennong Herbal Classic and believed to be able to activate blood, move “Qi” (vital energy) and alleviate painful conditions (Commission, 2015). Pharmacological studies display the multiple therapeutic effects of RC, including its anti-depression and anti-anxiety, anti-arrhythmia, anti-myocardial infarction, cerebral ischemia reperfusion (I/R) injury protection, anti-thrombosis, liver protection, anti-inflammation, and anticancer effects (Tian et al., 2020). Previous studies have revealed that separate or combined application of RC extracts can provide protection for the myocardium (Wu et al., 2007a; Xue et al., 2013; Li et al., 2020). However, the fact that the mechanism is unknown prevents extensive application of RC worldwide, hence the need for a precise evaluation.

**FIGURE 1:**
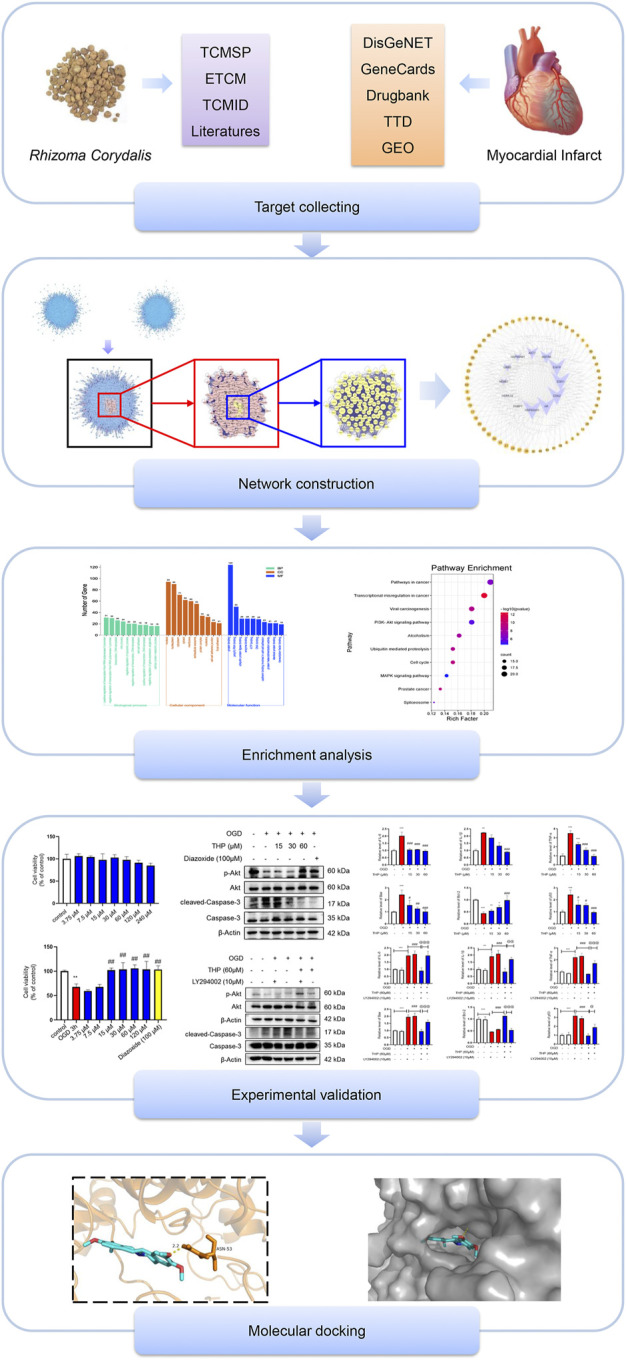
Diagram of the study strategy. Flowchart showing the combination of network pharmacology technology, experimental validation, and molecular docking used in this study.

Network pharmacology is an approach based on network construction and analysis technology and can systematically integrate and analyze information about bioactive components and the potential targets of components and diseases (Boezio et al., 2017). Recently, network pharmacology has been utilized to explore traditional Chinese medicine (TCM) in depth and has contributed to its modernization (Wang et al., 2021). In the present study, a network pharmacology approach, combined with specific experimental validation, was used to comprehensively elucidate the effect, and explore the molecular mechanism of RC on MI (a diagram of the study strategy is shown in Figure 1). This study provides a scientific basis for understanding the effect and mechanism of RC against MI and may suggest a novel therapeutic approach for MI.

## Materials and Methods

### Screening for the Bioactive Components of RC

The bioactive components of RC were filtered from the following databases: the Traditional Chinese Medicine Systems Pharmacology (TCMSP) Database (Ru et al., 2014), the Encyclopedia of Traditional Chinese Medicine (ETCM) Database (Xu et al., 2019) and the Traditional Chinese Medicine Integrated Database (TCMID) (Huang et al., 2018). RC is mainly taken orally, thus oral bioavailability (OB) and drug-likeness (DL) were used as filter conditions. The filter conditions were OB equal to or greater than 30% and DL equal to or greater than 0.18 (Li et al., 2019a). Components in the ETCM database for which the drug-likeness grading was moderate and good were screened. Components that met the conditions in the three databases were defined as bioactive components. All chemical information about the components, including molecular formulas, molecular weight, and 3D structures, was obtained from the PubChem database (Kim et al., 2019).

### Obtaining Potential Targets of RC and Identification of MI-Related Targets

Potential targets of bioactive components of RC were obtained from the PharmMapper database (Wang et al., 2017a) and human protein targets were selected. The MI-related targets were obtained from the following databases: DisGeNET (Pinero et al., 2020), GeneCards (Stelzer et al., 2016), DrugBank (Wishart et al., 2018), Therapeutic Target Database (TTD) (Yang et al., 2016), and Gene Expression Omnibus (GEO) datasets (Barrett et al., 2007). We used “myocardial infarction” as the search term, and the organism was restricted to *Homo sapiens*. The UniProtKB database was used to unify and standardize the targets (UniProt, 2015).

### Network Construction

The Cytoscape plugin BisoGenet was utilized to construct a protein-protein interaction (PPI) network of RC potential targets together with MI-related targets (Martin et al., 2010), and topological analysis was conducted by the Cytoscape plugin CytoNCA (Tang et al., 2015). The central PPI network was acquired after filtering twice by analyzing topological parameters degree centrality (DC), betweenness centrality (BC), closeness centrality (CC), eigenvector centrality (EC), local average connectivity-based method centrality (LAC), and network centrality (NC). The PPI network, component-target network, and Kyoto Encyclopedia of Genes and Genomes (KEGG) network were visualized using Cytoscape software.

### Enrichment Analysis

To explore the biological processes of core targets, Gene Ontology (GO) biological function and KEGG pathway enrichment analyses were performed with the online tool DAVID Bioinformatics Resources 6.8 (Chen et al., 2017). The screening criteria were set as *p* < 0.05, and the species was limited to *H. sapiens*.

### Molecular Docking

The crystal structure of core targets (macromolecules) was sought in the Protein Data Bank, and the structure (in SDF format) of the ligand (component) was retrieved from the PubChem database. We then changed the format of the molecules to pdb using OpenBabel software, and utilized AutoDockTools (version 1.5.6) for molecular docking. PyMOL software was then employed to display the docking model. All websites of the databases used in this study are listed in Table 1.

**TABLE 1:** The websites of the databases used in this study.

Database	Website
TCMSP	http://tcmspw.com/
ETCM	http://www.tcmip.cn/ETCM/index.php/Home/Index/
TCMID	http://www.megabionet.org/tcmid/
PubChem	https://pubchem.ncbi.nlm.nih.gov/
PharmMapper	http://lilab.ecust.edu.cn/pharmmapper/
DisGeNET	https://www.disgenet.org/
GeneCards	https://www.genecards.org/
DrugBank	https://www.drugbank.ca/
TTD	http://db.idrblab.net/ttd/
GEO	https://www.ncbi.nlm.nih.gov/geo/
UniProtKB	https://www.uniprot.org/
DAVID	https://david.ncifcrf.gov/
Protein Data Bank	https://www.rcsb.org/

### Chemicals and Reagents

Tetrahydropalmatine (THP, B74569) (purity ≥ 98%, HPLC) was purchased from Shanghai Yuanye Biotechnology Co., Ltd. (Shanghai, China). The THP powder was dissolved in dimethyl sulfoxide (DMSO, Sigma, St. Louis, MO, United States). Diazoxide (01131863) was provided by Adamas-beta (Basel, Switzerland), and LY294002 (S1105) was provided by Selleck (Shanghai, China).

### Cell Culture and Treatment

H9c2 cells were plated into a dish (35 mm) at a density of 1 × 10^6^ cells and then maintained in Dulbecco’s modified Eagle’s medium (DMEM) containing 10% fetal bovine serum (FBS). The cells were grown at 37°C in humidified 5% CO_2_. Before the subsequent experiments, a serum-free conditioned medium was used for cell culture.

### Cell Viability Assay

A Cell Counting Kit-8 (CCK-8) assay kit (GLPBIO, California, United States) was used to examine cell activity. Briefly, H9c2 cells were seeded into 96-well plates. After treatment, the cells were incubated with 10 μl CCK-8 solution per well for 2 h at 37°C, and after incubation, the absorbance value at 450 nm was measured using a microplate reader (Thermo Varioskan LUX, MA, United States).

### Western Blot Analysis

Western blot assays were performed as we previously reported (Li et al., 2016b; Li et al., 2019b). Primary antibodies against phospho-Akt (Ser473) (Cat# 4060), Akt (Cat# 4691), and caspase-3 (Cat# 9692) were provided by Cell Signaling Technology (Danvers, Massachusetts, United States). A primary antibody against cleaved-caspase-3 (bsm-33199M) was provided by Bioss (Beijing). Anti-β-actin antibody (GB12001) was provided by Servicebio (Wuhan). Equivalent amounts of protein (20 μg protein extracts) were separated by 8%–12% sodium dodecyl sulfate polyacrylamide gel electrophoresis (SDS-PAGE) and then transferred to polyvinylidene fluoride (PVDF) membranes (Millipore, United States). The PVDF membranes were incubated overnight with the primary antibodies at 4°C.

After overnight incubation, appropriate horseradish peroxidase (HRP)-conjugated secondary antibodies were added and incubated at room temperature for 1 h. The protein level signals were visualized using ECL-enhanced chemiluminescence (Tanon, Shanghai), and the band intensities were quantified by ImageJ software (Bio-Rad).

### Quantitative Real-Time Polymerase Chain Reaction

The H9c2 cells were lysed with Accurate Biology RNAex Pro Reagent (AG21102, Accurate Biology, Hunan, China) to extract total RNA. Then 2 μg of RNA was reversely transcribed to cDNA with the Evo M-MLV RT Kit (Accurate Biotechnology, Human, China). The mRNA levels were determined with a SYBR Green qPCR kit (Accurate Biotechnology, Human, China) by QuantStudio 5 Real-Time PCR instrument (Thermo Fisher Scientific, MA, United States). Rat-specific primers for BCL2 Associated X (Bax), B cell lymphoma-2 (Bcl-2), p53, interleukin-6 (IL-6), interleukin-1 beta (IL-1β), tumor necrosis factor-α (TNF-α), and β-actin were synthesized by Sangon Biotech (Shanghai) (Table 2). All semiquantitative and quantitative real-time polymerase chain reaction (RT-qPCR) data were quantified with respect to β-actin. The results were calculated by the 2^−ΔΔCt^ method.

**TABLE 2:** Nucleotide sequences of the gene-specific primers used for RT-qPCR.

Primer	Sequences
Bax	Forward: 5′-TGG​GAT​GGC​CTC​CTT​TCC​TA-3′
	Reverse: 5′-TTC​CCC​GTT​CCC​CAT​TCA​TC-3′
Bcl-2	Forward: 5′-TGG​AGA​GCG​TCA​ACA​GGG​AGA​TG-3′
	Reverse: 5' -GTG​CAG​ATG​CCG​GTT​CAG​GTA​C-3′
p53	Forward: 5′-CCT​TAC​CAT​CAT​CAC​GCT​GGA​AGA​C-3′
	Reverse: 5′-AGG​ACA​GGC​ACA​AAC​ACG​AAC​C-3′
IL-6	Forward: 5′-ACT​TCC​AGC​CAG​TTG​CCT​TCT​TG-3′
	Reverse: 5′-TGG​TCT​GTT​GTG​GGT​GGT​ATC​CTC-3′
IL-1β	Forward: 5′-TGC​AGG​CTT​CGA​GAT​GAA​C-3′
	Reverse: 5′-GGG​ATT​TTG​TCG​TTG​CTT​GTC-3′
TNF-α	Forward: 5′-CTT​CTG​TCT​ACT​GAA​CTT​CGG​G-3′
	Reverse: 5′-CTA​CGG​GCT​TGT​CAC​TCG-3′
β-Actin	Forward: 5′-TCG​TGC​GTG​ACA​TTA​AAG​AG-3′
	Reverse: 5′-ATT​GCC​GAT​AGT​GAT​GAC​CT-3′

### TdT-Mediated dUTP Nick-End Labeling Staining

A one Step TdT-Mediated dUTP Nick-End Labeling (TUNEL) Apoptosis Assay Kit (Beyotime, China) was used to measure cell apoptosis. H9c2 cells were washed with phosphate buffered saline (PBS) three times and fixed with 4% paraformaldehyde for 10 min. After washing with PBS three times, H9c2 cells were incubated with 50 μl of TUNEL detection solution at 37°C for 1 h in the dark and then incubated with 4′,6-diamidino-2-phenylindole (DAPI) for 10 min to visualize nuclei. The green spectrum and blue spectrum were used to detect TUNEL-positive cells and nuclei, respectively. Finally, the cells were photographed by confocal scanning microscopy (Leica TCS SP8, Leica, Germany). TUNEL-positive cells were quantified and analyzed from four different views. Data were expressed as the percentage of the number of TUNEL-positive nuclei in the total number of nuclei detected by DAPI staining. ImageJ software was used to calculate the number of TUNEL positive cells.

### Statistical Analysis

The data in this study are presented as mean ± SD from three independent replicates. One-way analysis of variance (ANOVA) with the Bonferroni post-hoc test was performed among the multiple groups. In all cases, *p* < 0.05 was considered statistically significant.

## Results

### Target Prediction and Analysis

To collect the components of RC, we searched three databases, and in all, 60 bioactive components were collected by filtering the TCMSP, ETCM, and TCMID databases by the limitations of OB ≥ 30% and DL ≥ 0.18. The active ingredients mainly included berberine, sitosterol, tetrahydropalmatine, corydaline, and quercetin. The information about the components is listed in Supplementary Table S1. The potential targets were predicted according to the PharmMapper database, which was based on a large-scale reverse pharmacophore mapping strategy. Finally, we obtained 431 potential targets after removing duplicates. The component-target network is shown in Supplementary Figure S1.

To facilitate statistics, we set screening conditions for MI targets derived from corresponding databases. After taking the third quartile value, a relevance score ≥ 3.66 was set as the threshold for targets from the GeneCards database, and a score ≥ 0.1 was set as the threshold for those from the DisGeNET database. While searching GEO datasets, we selected the reference series GSE48060 to obtain the differentially expressed genes between normal cardiac function controls and first-time MI patients (Suresh et al., 2014). The filter conditions were adjusted as *p* < 0.05 and absolute fold change of 1.5 or greater. After deleting duplicates, 1,131 targets were obtained in total.

### Protein-Protein Network Analysis

**FIGURE 2:**
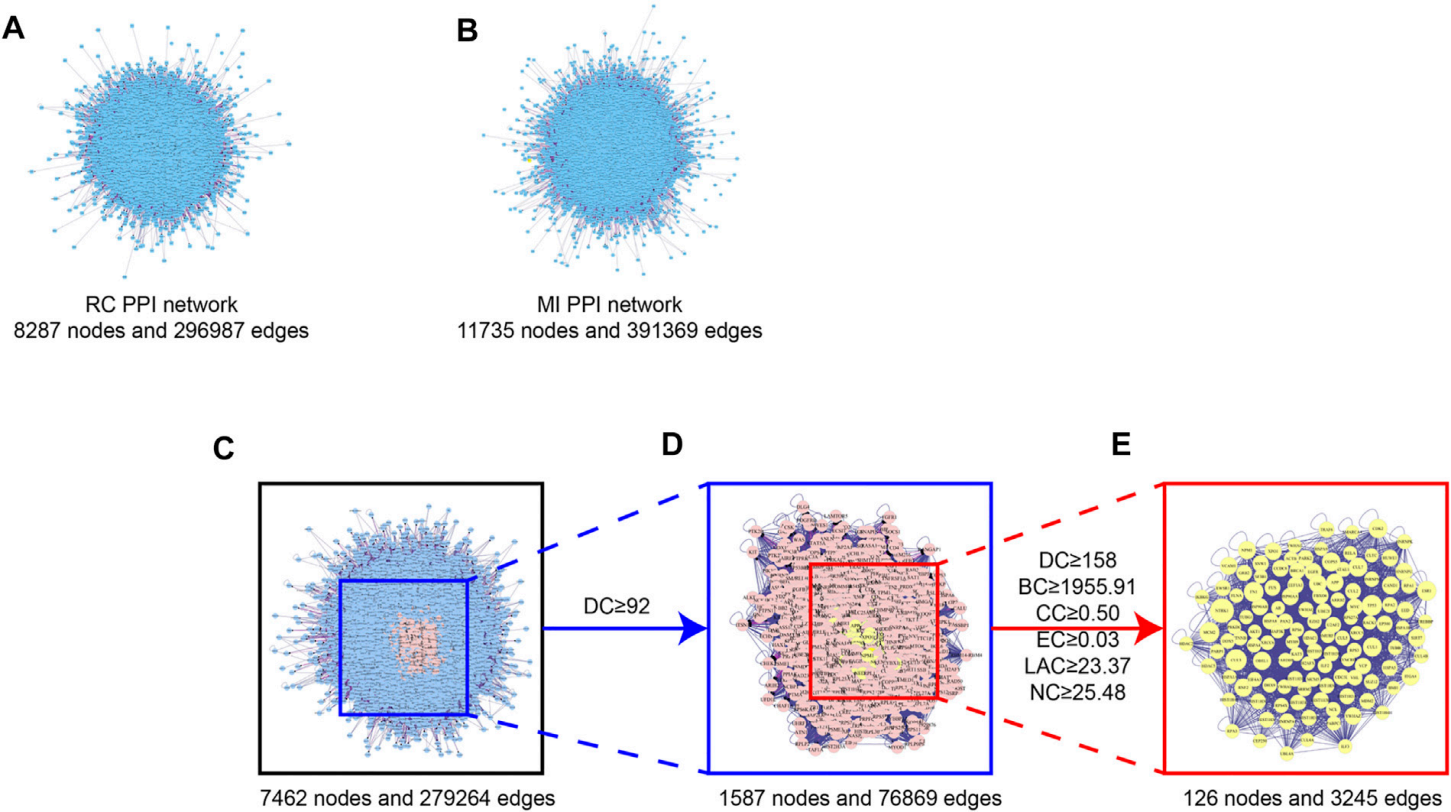
Topological screening process of PPI network. **(A)** PPI network of the potential targets of the RC ingredients. 8,287 nodes represent the interactive proteins of RC, 2,96,987 edges represent the interactive relationship. **(B)** PPI network of the MI-related targets. 11,735 nodes represent the interactive proteins of MI, 3,91,369 edges represent the interactive relationship. **(C)** PPI network of merged RC and MI. 7,462 nodes represent the interactive proteins of the merged network, 2,79,264 edges represent the interactive relationship. **(D)** PPI network of merged RC and MI with DC ≥ 92. 1,587 nodes represent the interactive proteins of the merged network with DC ≥ 92, 76,869 edges represent the interactive relationship. **(E)** Central PPI network of merged RC and MI. 126 nodes represent the core proteins of the merged network, 3,245 edges represent the interactive relationship.

**FIGURE 3:**
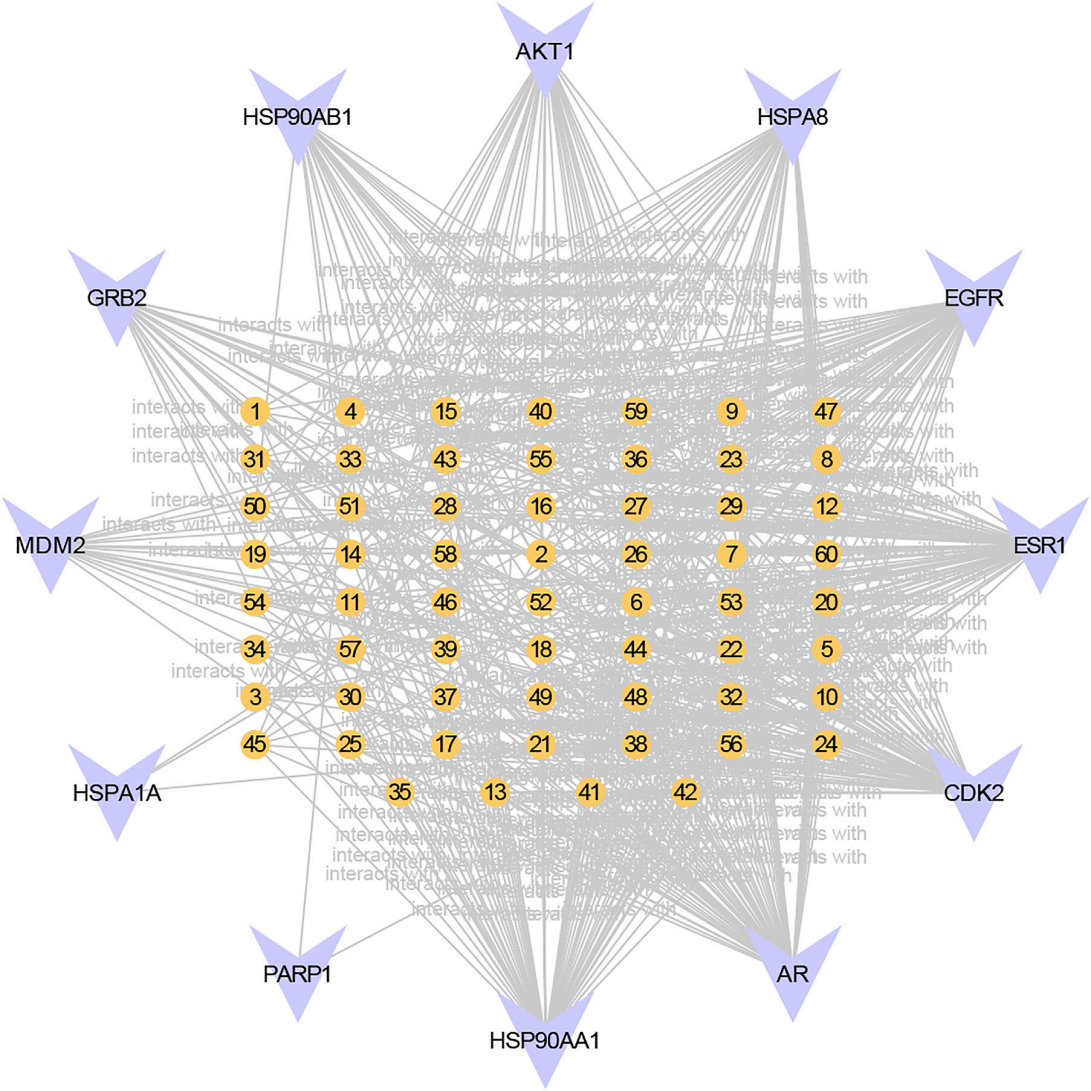
Component-core target network of RC and 12 core targets. 12 of the 126 core targets were RC-targets that correlated with 60 total bioactive components. The orange nodes represent the potential active ingredients, and the violet nodes represent the core targets.

We used the Cytoscape plugin BisoGenet to construct a PPI network of RC potential targets (8,287 nodes and 296,987 edges) (Figure 2A) and MI-related targets (11,735 nodes and 391,369 edges) (Figure 2B). To identify a comprehensive RC-MI-target network, we merged the two PPI networks and there were 7,462 nodes and 279,264 edges in total in the new PPI network (Figure 2C). From topological analysis conducted by the plugin CytoNCA, we performed filtration twice. The first screening condition is that DC is greater than and equal to double median (DC ≥ 92) (Figure 2D), and the second screening condition is that DC, BC, EC, LAC, and NC are greater than and equal to double median (DC ≥ 158, BC ≥ 1955.51, EC ≥ 0.03, LAC ≥ 23.37, and NC ≥ 25.48), and CC is greater than and equal to median (CC ≥ 0.50). Ultimately, a central PPI network with 126 nodes and 3,245 edges was obtained (Figure 2E). The 126 nodes represented 126 core targets associated with both RC and MI, and the information about the 126 core targets is listed in Supplementary Table S2. We also performed a component-core target network and found that 12 of the 126 core targets, including androgen receptor (AR), estrogen receptor 1 (ESR1), cyclin-dependent kinase 2 (CDK2), heat shock protein 90 kDa alpha A1 (HSP90AA1), heat shock 70 kDa protein 8 (HSPA8), epidermal growth factor receptor (EGFR), AKT1, poly (ADP-ribose) polymerase 1 (PARP1), growth factor receptor-bound protein 2 (GRB2), mouse doubleminute 2 (MDM2), heat shock protein 90 kDa alpha (cytosolic), class B member 1 (HSP90AB1), and heat shock 70 kDa protein 1A (HSPA1A), could be regulated by the 60 bioactive components (Figure 3; Supplementary Table S3).

### GO Analysis and KEGG Analysis of the Core Targets

**FIGURE 4:**
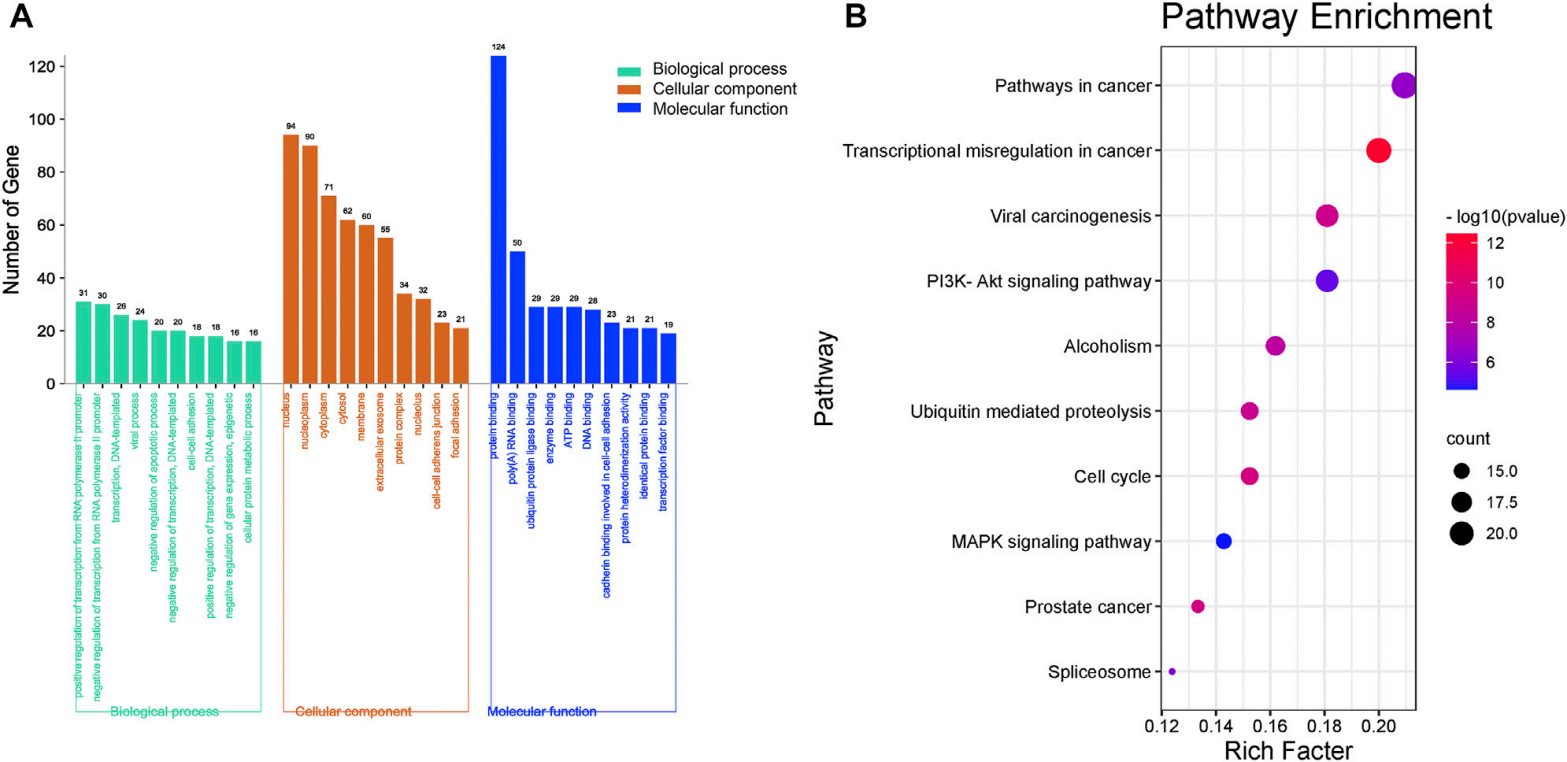
Enrichment of GO and KEGG analysis of the 126 key genes targeted by RC against MI. **(A)** The GO enrichment results of biological processes, cellular components, and molecular function. **(B)** Enriched KEGG pathways of potential key targets. The top 10 major enriched pathways are shown.

To identify the biological function of the core targets, GO and KEGG enrichment analyses of the 126 core targets were performed using the DAVID online tool. Ultimately, a total of 126 targets were analyzed by GO, while 105 targets were enriched in KEGG pathways. The top 10 results of biological process analysis, cellular component analysis, and molecular function analysis of GO function and the top 10 major KEGG enriched pathways are shown in Figure 4. The results suggest that 126 core targets could regulate biological processes involving positive regulation of transcription from RNA polymerase II promoter (*p* = 0.000000000020993, 31 targets/981), negative regulation of transcription from RNA polymerase II promoter (*p* = 0.000000000000054, 30 targets/720), transcription, DNA-templated (*p* = 0.004630802658299, 26 targets/1955), viral process (*p* = 0.000000000000000, 24 targets/299), negative regulation of apoptotic process (*p* = 0.000000001107359, 20 targets/455), negative regulation of transcription, DNA-templated (*p* = 0.000000005053472, 20 targets/499), cell-cell adhesion (*p* = 0.000000000017396, 18 targets/271), positive regulation of transcription, DNA-templated (*p* = 0.000000270675033, 18 targets/515), negative regulation of gene expression, epigenetic (*p* = 0.000000000000000, 16 targets/50) and cellular protein metabolic process (*p* = 0.000000000000010, 16 targets/118).

The top 10 cellular components were nucleus (*p* = 0.000000000000000, 94 targets/5,415), nucleoplasm (*p* = 0.000000000000000, 90 targets/2,784), cytoplasm (*p* = 0.000000000132580, 71 targets/5,222), cytosol (*p* = 0.000000000000005, 62 targets/3,315), membrane (*p* = 0.000000000000000, 60 targets/2,200), extracellular exosome (*p* = 0.000000000000097, 55 targets/2,811), protein complex (*p* = 0.000000000000000, 34 targets/412), nucleolus (*p* = 0.000000000000013, 32 targets/857), cell-cell adherens junction (*p* = 0.000000000000000, 23 targets/323) and focal adhesion (*p* = 0.000000000002406, 21 targets/391).

The major molecular functions were protein binding (*p* = 0.000000000000000, 124 targets/8,785), poly(A) RNA binding (*p* = 0.000000000000000, 50 targets/1,129), ubiquitin protein ligase binding (*p* = 0.000000000000000, 29 targets/287), enzyme binding (*p* = 0.000000000000000, 29 targets/333), ATP binding (*p* = 0.000003513330142, 29 targets/1,495), DNA binding (*p* = 0.000078465083276, 28 targets/1,674), cadherin binding involved in cell-cell adhesion (*p* = 0.000000000000000, 23 targets/290), protein heterodimerization activity (*p* = 0.000000000209051, 21 targets/465), identical protein binding (*p* = 0.000000600209776, 21 targets/749) and transcription factor binding (*p* = 0.000000000003245, 19 targets/284).

**FIGURE 5:**
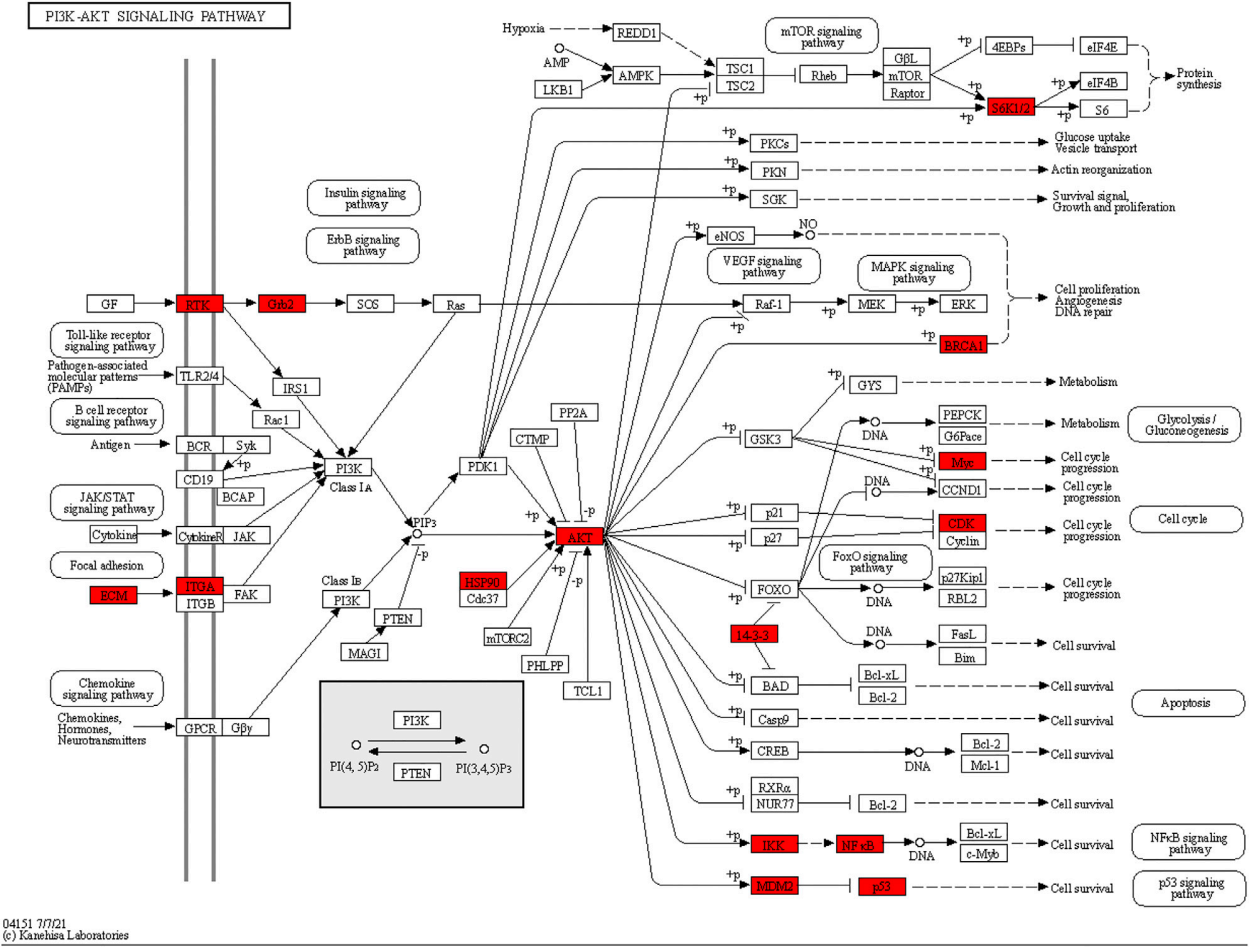
Core targets enriched in the PI3K/Akt pathway. The red nodes represent 15 core targets enriched in the PI3K/Akt pathway.

As the KEGG analysis of the core target results showed, among the 10 pathways, we found that the 126 core targets were primarily involved in the phosphatidylinositol 3-kinase (PI3K)/protein kinase B (PKB, also called Akt) signaling pathway (*p* = 0.000003284827250, 19 targets/345). As Figure 3 shows, most components had the potential to regulate Akt. The PI3K/Akt pathway was enriched with 15 core targets, as shown in Figure 5.

### Results of Molecular Docking Analysis

**FIGURE 6:**
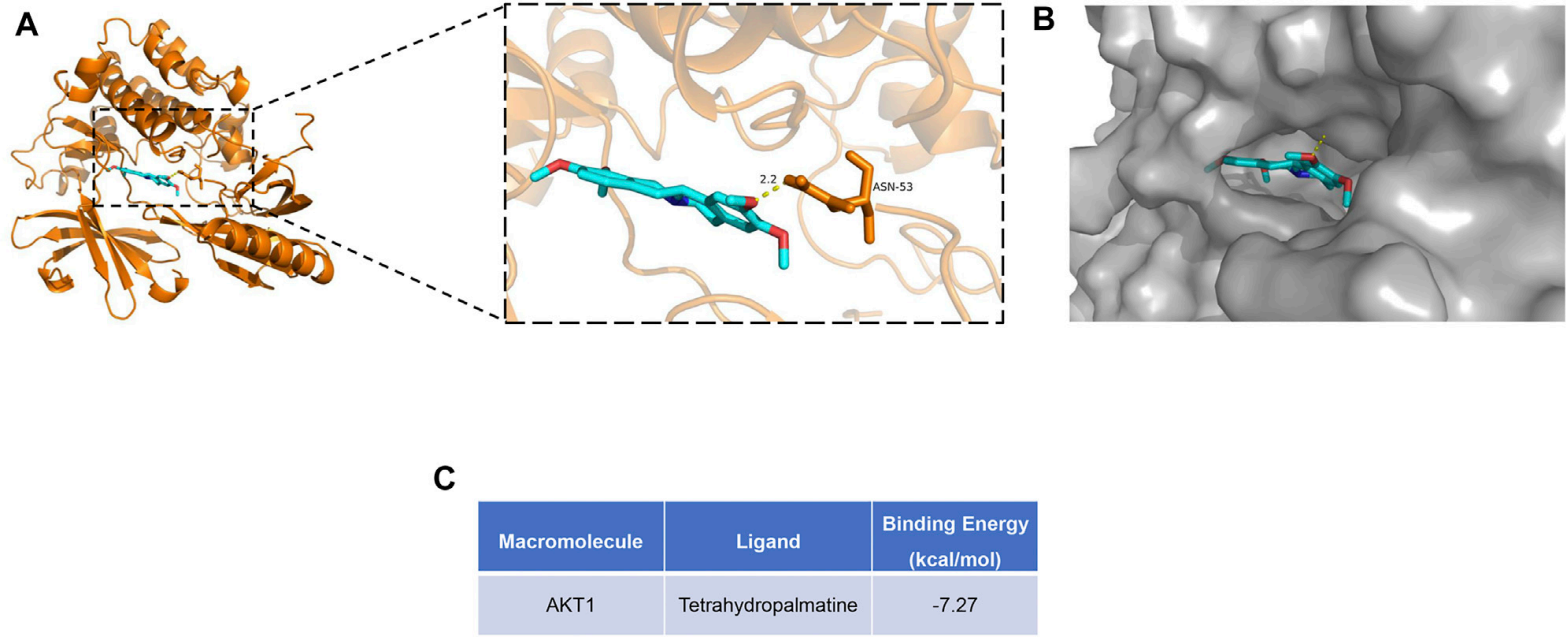
Molecular docking diagram of Akt with THP. **(A)** Structure diagram of Akt docked with THP. Yellow lines represent hydrogen bond. **(B)** Surface diagram of Akt docked with THP. **(C)** The binding energy for THP docked into the Akt crystal structure.

Among these bioactive components (Figure 3), some were dedicated to pain relief or cancer treatment (Gao et al., 1994; Xu et al., 2012; Chen et al., 2016; Wang et al., 2016; Wang et al., 2019), while berberine, coptisine, THP, palmatine, and quercetin were reported to be cardioprotective (Kim et al., 2009; Guo et al., 2013; Zhu et al., 2020; Albadrani et al., 2021). In this study, we conducted molecular docking of the 5 components with AKT1, the core target related to the PI3K/Akt pathway. The results are displayed in Figure 6 and Supplementary Figure S2. Among the 5 components, THP had the lowest binding energy with AKT1. A previous study suggested that THP plays a beneficial role in MI-induced heart failure and MI/RI (Wu et al., 2007a), while its effect on MI remains elusive. THP was recently reported to modulate the PI3K/Akt/mTOR pathway to protect against limb ischemia/reperfusion injury (Wen et al., 2020). Therefore, we speculated that THP may alleviate MI injury by regulating the PI3K/Akt pathway. To this end, we conducted molecular docking. A docking diagram of Akt (PDB 6HHG) and THP (CAS: 6024-85-7) is displayed in Figures 6A,B. Hydrogen bonds were formed between THP and amino acid residues Asn 53 in the crystal structure of Akt. The binding affinity of THP with the Akt crystal structure was −7.12 kcal/mol (Figure 6C). This result demonstrated that THP has a strong binding capacity with Akt. Thus, THP was used for the subsequent experimental validation.

### THP Protected H9c2 Cells Against OGD-Induced Injury

**FIGURE 7:**
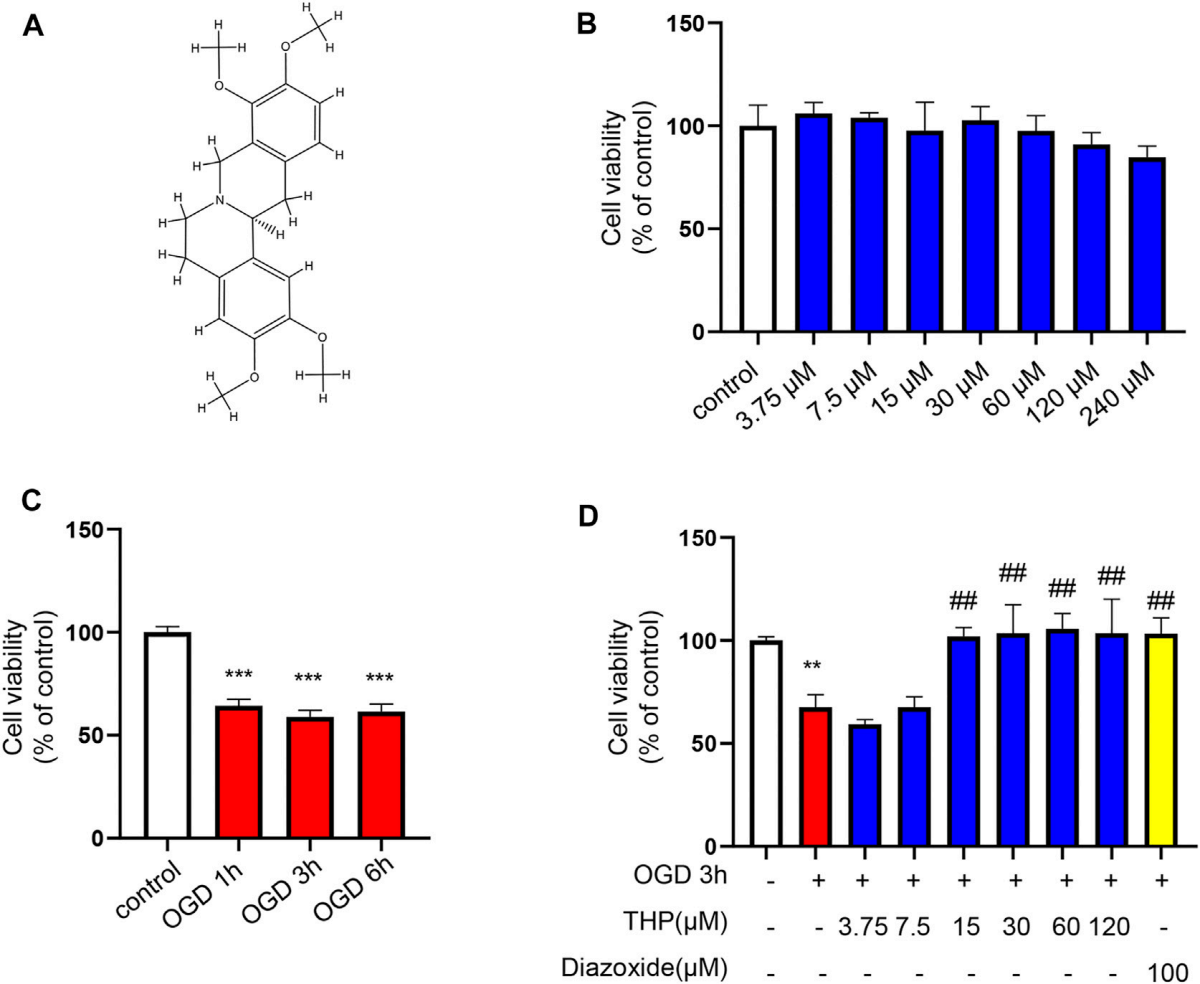
THP had no cellular toxicity on H9c2 cells under 240 μM and protected H9c2 against OGD-induced injury. **(A)** Chemical structure of THP. **(B)** H9c2 cells were incubated with THP for the indicated concentrations ranging from 3.75 to 240 μM for 24 h. **(C,D)** The effect of THP on the OGD-induced H9c2 cells. H9c2 cells were treated with different concentrations of THP or diazoxide for 48 h following OGD exposure. Then the H9c2 cells were cultured under OGD condition for the indicated time. A CCK-8 assay was used to determine cell viability. Data are presented as the mean ± SD. **p < 0.01, ***p < 0.001 vs. control, ^##^ *p* < 0.01 vs. OGD. *n* = 3 (three independent replicates).

To elucidate the efficacy of THP (Chemical structure of THP showed in Figure 7A) against MI, we first assessed the cytotoxicity of THP on the growth of H9c2 cells. The results showed that in resting cells, THP had no toxicity to H9c2 cells below 240 μM (Figure 7B). Under ischemic conditions, a reduction in glucose and oxygen causes damage to cardiomyocytes. Among the various methods for simulating the pathological process of ischemia *in vitro*, oxygen and glucose deprivation insult has been widely used as a classic approach. Therefore, to investigate the cardioprotective effect of THP, we constructed an OGD model in H9c2 cells. H9c2 cells were exposed to OGD insult for the indicated times, and cell viability was determined using a CCK8 assay kit. As Figure 7C shows, after OGD for 1, 3, or 6 h, the viability of H9c2 cells was significantly reduced. Because cell injury was the most obvious at 3 h, experimental measurements were taken after the cells were treated for 3 h. We then treated H9c2 cells with various doses of THP and the positive control drug diazoxide for 48 h before being exposed to OGD. As shown in Figure 7D, THP treatment (15 μM or more) protected H9c2 cells against OGD-induced injury. Thus, 15, 30, and 60 μM THP were chosen for the following experiments.

### THP Decreased OGD-Induced Apoptosis in H9c2 Cells

**FIGURE 8:**
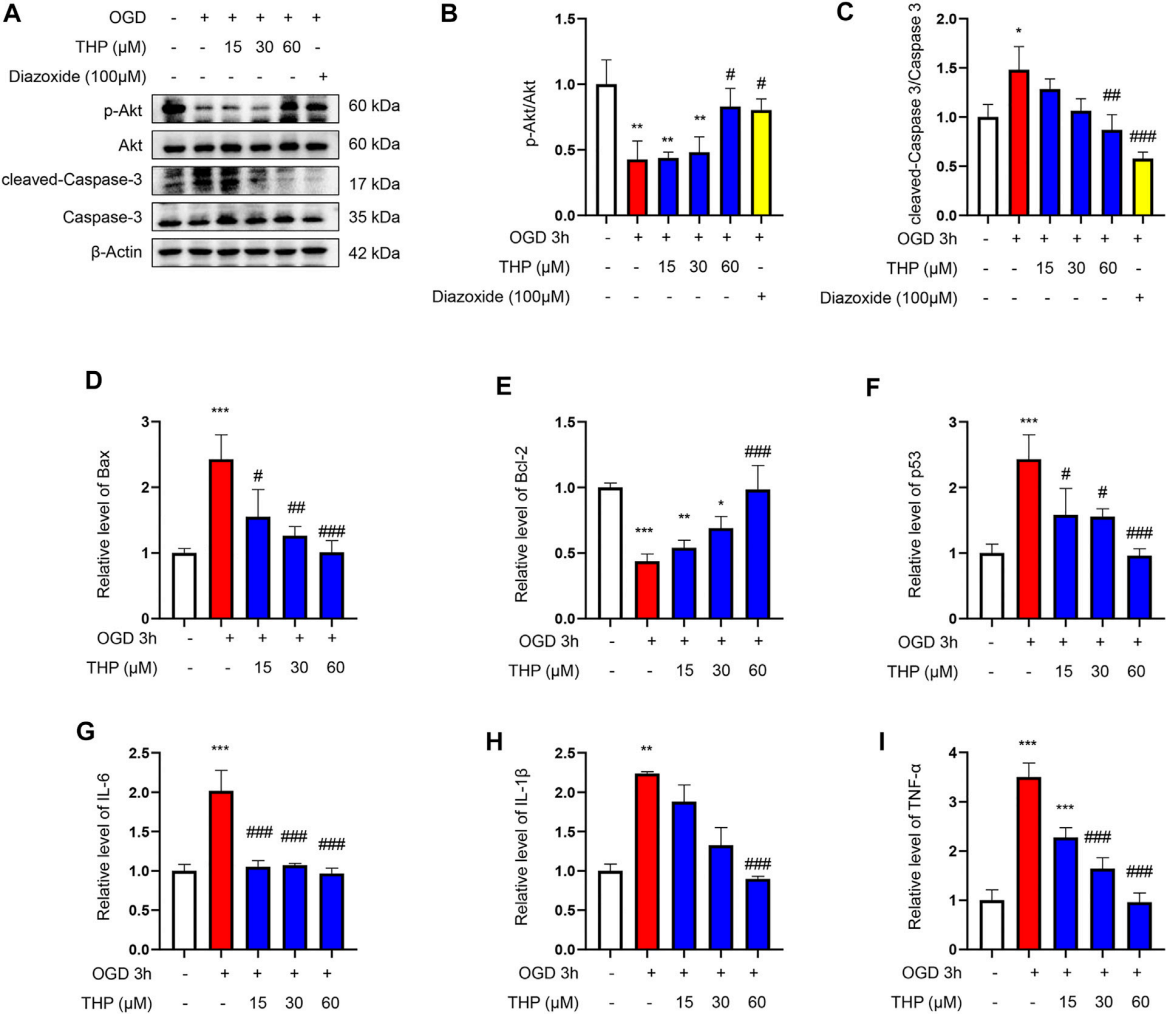
THP was able to suppress apoptosis and the expressions of inflammatory factors induced by OGD in H9c2 cells. The cultured cells were treated with different concentrations of THP or diazoxide and stimulated with OGD for 3 h **(A–C)** The protein expressions of p-Akt, total Akt, cleaved caspase-3, and caspase-3. **(D–F)** The mRNA expressions of apoptosis factors (Bax, Bcl-2, and p53). **(G–I)** The mRNA expressions of inflammatory factors (IL-6, IL-1β, and TNF-α). Data are shown as the mean ± SD. *p < 0.05, **p < 0.01, ***p < 0.001 vs. control; ^#^ *p* < 0.05, ^##^ *p* < 0.01, ^###^ *p* < 0.001 vs. OGD. *n* = 3 (three independent replicates).

As shown in Figures 8A,C, OGD notably elevated the cell apoptosis level, as indicated by the increased protein level of cleaved caspase-3/caspase-3, as well as the increased mRNA level of Bax, and the reduced mRNA level of Bcl2 (Figures 8D,E). Moreover, the mRNA level of p53 was increased after OGD for 3 h (Figure 8F), and THP treatment significantly reduced OGD-induced apoptosis (Figures 8A–F). The anti-apoptosis effect of 60 μM THP seemed equal to that of 100 μM diazoxide. Our results showed that THP greatly decreased apoptosis of H9c2 cells after OGD exposure, supporting the protective effect of THP in MI injury.

### THP Decreased the OGD-Induced Expression of Inflammatory Factors

In the present study, OGD induced a series of inflammatory changes that mediated cardiomyocyte injury. The mRNA expression levels of inflammatory factors, including IL-6, IL-1β, and TNF-α, were significantly increased in the OGD group compared to the control group. However, THP treatment (15, 30, and 60 μM) significantly inhibited the overproduction of these inflammatory biomarkers compared to the OGD-induced group (Figures 8G–I). Collectively, THP was shown to significantly suppress the inflammatory process.

### THP Elevated Akt Phosphorylation

The results from KEGG enrichment showed that RC and THP were closely related to the PI3K/Akt signaling pathway against MI and that most components could regulate Akt. Therefore, to confirm whether THP exhibits a protective effect via the regulation of Akt, the phosphorylation level and protein expression of Akt were detected. As shown in Figure 8A, OGD significantly decreased Akt phosphorylation (Ser473). Moreover, THP strongly elevated Akt phosphorylation in a dose-dependent manner, without affecting the expression of total Akt. These data suggest that THP might suppress MI injury by regulating the Akt signaling pathway.

### THP Exhibited Anti-Apoptotic and Anti-Inflammatory Effects *via* the PI3K/Akt Signaling Pathway

**FIGURE 9:**
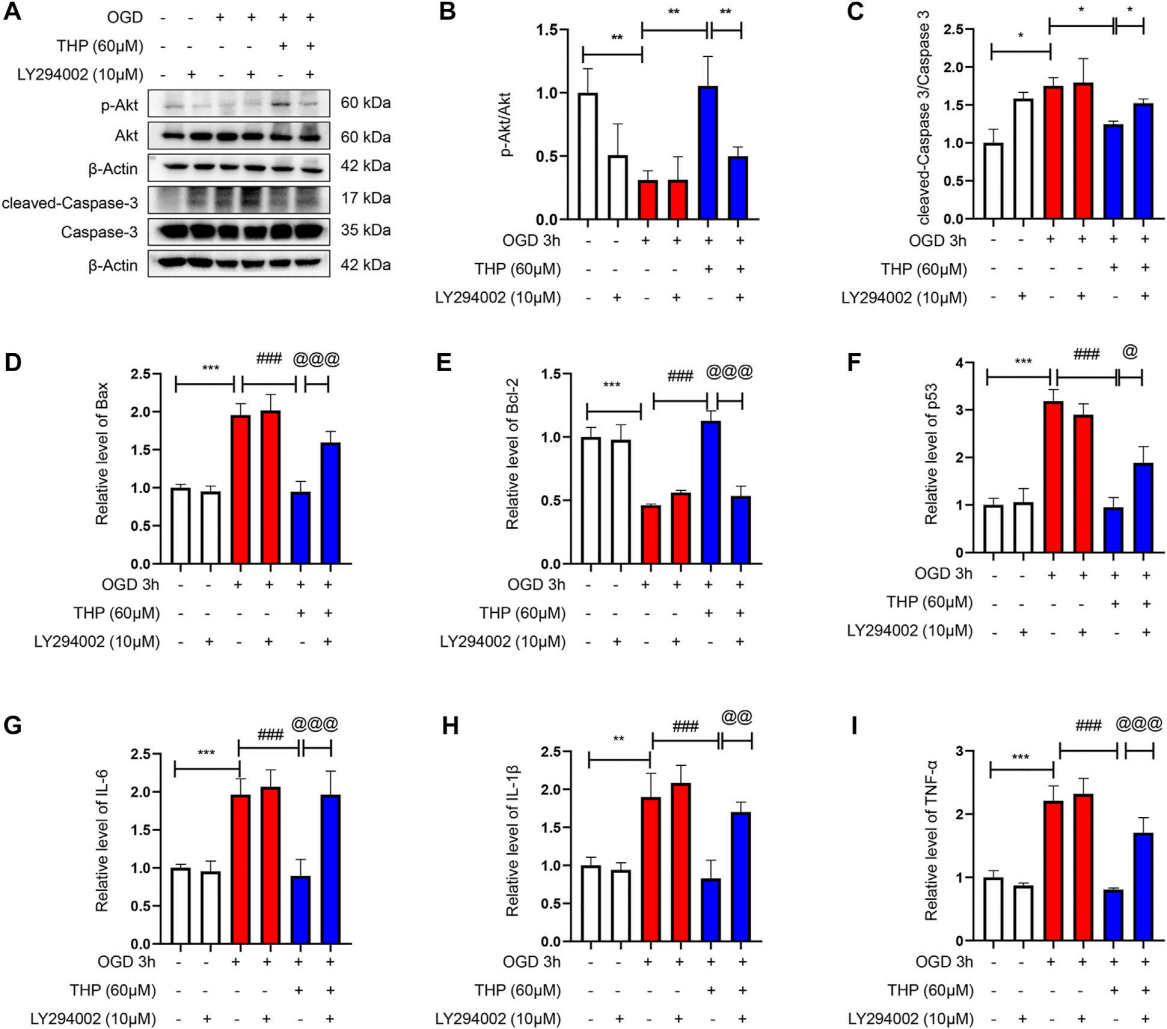
THP exhibited anti-apoptotic effect and suppressed the expression of inflammatory factors via regulating the PI3K/Akt signaling pathway. H9c2 cells were incubated with LY294002 (10 μM, diluted in DMSO), an inhibitor of the PI3K/Akt pathway or treated with THP (60 μM) for 48 h, and then the cells were stimulated with OGD. **(A–C)** The protein expressions of p-Akt, total Akt, cleaved caspase-3, and caspase-3. **(D–F)** The mRNA expressions of apoptosis factors (Bax, Bcl-2, and p53). **(G–I)** The mRNA expressions of inflammatory factors (IL-6, IL-1β, and TNF-α). Data are shown as the mean ± SD. *p < 0.05, **p < 0.01, ***p < 0.001 vs. control; ^#^ *p* < 0.05, ^##^ *p* < 0.01, ^###^ *p* < 0.001 vs. OGD, ^@^ *p* < 0.05, ^@@^ *p* < 0.01, ^@@@^ *p* < 0.001 vs. OGD + THP. *n* = 3 (three independent replicates).

**FIGURE 10:**
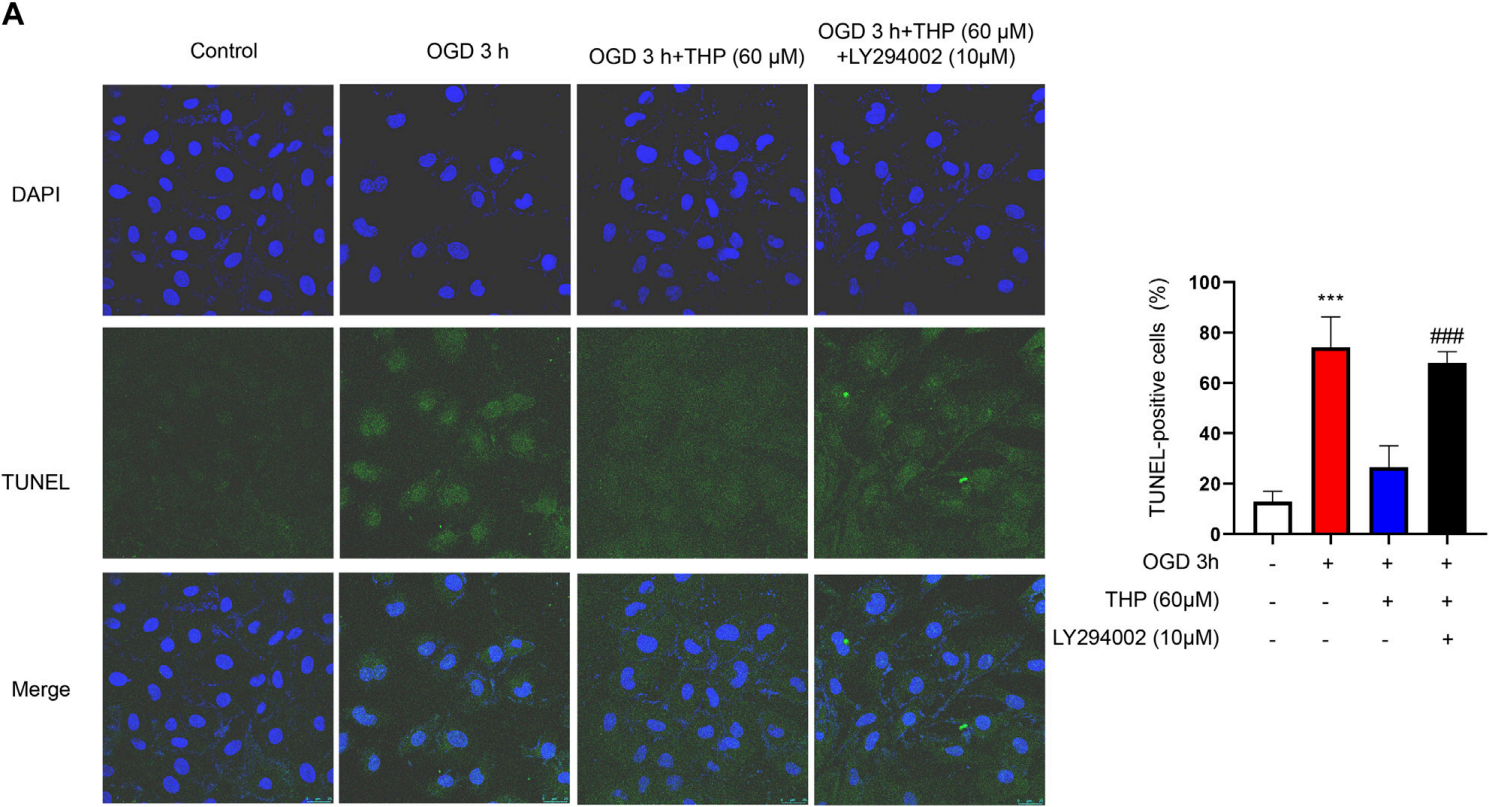
THP decreased H9c2 cells apoptosis by activating the PI3K/Akt signaling pathway. H9c2 cells were incubated with LY294002 (10 μM, diluted in DMSO) or treated with THP (60 μM) for 48 h, and then the cells were stimulated with OGD. **(A)** H9c2 cells apoptosis was assessed through TUNEL assay (scale bar = 25 μm).

The PI3K/Akt signaling pathway is significant in defending against myocardial infarction damage (Feng et al., 2020; Ruan et al., 2020). According to previous experiments, we ascertained that THP plays an important role in inhibiting apoptosis and inflammation. To further clarify the significance of the PI3K/Akt signaling pathway in this process, we used LY294002, an inhibitor of the PI3K/Akt pathway, to block the PI3K/Akt pathway. In our experiment we found that the upregulated cleaved caspase-3/caspase-3, Bax, and p53 that had been induced by OGD were downregulated by THP, and the downregulated Bcl2, induced by OGD, was upregulated by THP, but these effects were diminished by LY294002 (10 μM) treatment (Figures 9A–F). Moreover, LY294002 treatment also prevented the anti-inflammatory effect of THP, as indicated by the reduced mRNA levels of IL-6, IL-1β, and TNF-α (Figures 9G–I). In addition, the TUNEL assay results showed that OGD induced a marked elevation in the number of TUNEL-positive cells (green nuclear staining), whereas THP treatment reduced the number of TUNEL-positive cells. However, LY294002 treatment reversed the anti-apoptotic effect of THP in H9c2 cells stimulated with OGD (Figure 10). Accordingly, these findings demonstrate that THP protected H9c2 cells from OGD-induced apoptosis by activating the PI3K/Akt pathway.

## Discussion

MI is a severe disease with high motility that places a heavy burden on individuals and society. The current mechanisms of MI include cell death, mitochondrial dysfunction, inflammatory response, oxidative stress, and ATP depletion (Frangogiannis, 2014; Frangogiannis, 2015; Kurian et al., 2016). In addition, MI can lead to various complications, such as arrhythmia, heart failure, and cardiac rupture (Gong et al., 2021). TCM, including individual herbs or combination formulas that contain multiple compounds, may have positive pharmacological benefits in the treatment of MI. RC is usually applied alone or in a formula in treating MI in China. RC can ameliorate symptoms and reduce the incidence of severe complications, although the specific mechanism remains unknown. In this study, a network pharmacology approach, accompanied by experimental validation and molecular docking analysis, was applied to explore the underlying pharmacological mechanism of RC in treating MI (Figure 1).

The chemical components of RC can be classified as alkaloids (including berberine, aporphine, opiates, isoquinoline, steroids, organic acid, carbohydrates, and others) (Tian et al., 2020). In this study, after screening RC in the TCMSP, ETCM, and TCMID databases with the standards of OB ≥ 30%, as well as DL ≥ 0.18, we filtered 60 bioactive components, of which many have been shown to be cardioprotective (Supplementary Figure S1; Supplementary Table S1). Quercetin can protect cardiomyocytes against ischemia, hypoxia or isoproterenol (ISO) insults due to its anti-inflammatory, antioxidant, and anti-apoptotic effects, and by partly regulating silent information regulatory factor 1 (SIRT1) and adenosine monophosphate-activated protein kinase (AMPK) pathways (Li et al., 2016a; Kumar et al., 2017; Guo et al., 2019; Tang et al., 2019). Emodin inhibit apoptosis and the inflammatory response to protect the myocardium from ischemia or hypoxia-induced injury (Wu et al., 2007b; Ye et al., 2019; Zhang et al., 2019). Palmatine can produce antioxidant and anti-inflammatory actions to reduce myocardial I/R injury by decreasing serum levels of creatine phosphokinase (CK), lactate dehydrogenase (LDH), and malonaldehyde (MDA), and also hinders decline in the activity of superoxide dismutase (SOD) and catalase (Kim et al., 2009). Berberine has anti-oxidant, anti-apoptosis, and anti-inflammation functions, promoting autophagy and attenuating endoplasmic reticulum (ER) stress to protect the myocardium from I/R injury by regulating the toll-like receptor 4 (TLR4) pathway, PI3K/Akt signaling pathway, SIRT1 pathway, Janus kinase 2 (JAK2)/signal transducer and activator of transcription 3 (STAT3) pathway, and hypoxia inducible factor-1α (HIF-1α) pathway (Liu et al., 2019; Zhu et al., 2020). Vanillic acid reduces infarct size and inhibits the apoptosis pathway by regulating caspase-9, caspase-3, and Bcl-2 (Prince et al., 2011; Stanely Mainzen Prince et al., 2015; Radmanesh et al., 2017). Coptisine reduces infarct size and protects cardiomyocytes from apoptosis and oxidative stress, partly through the Beclin-1/SIRT1 pathway and RhoA/Rho-associated kinase pathway (Gong et al., 2012; Guo et al., 2013; Wang et al., 2017b). Glaucine, dehydrocorydaline, canadine, tetrahydrocoptisine, and corydaline exhibit antiplatelet activity that strongly inhibits thrombin-induced platelet aggregation (Zhang et al., 2016). Allocryptopine exhibits an antiarrhythmic effect and can blunt the atrial late sodium current increase, reduce the current densities of the outward potassium current and slow the delayed rectifier potassium current (Fu et al., 2016; Dong et al., 2019). Protopine is reported to provide protection for rats with cerebral ischemic injury and suppress platelet aggregation and inflammatory response (Chia et al., 2006; Xiao et al., 2007; Bae et al., 2012; Alam et al., 2019). Additionally, protopine may be cardioprotective against MI according to the above effects. All of this evidence suggests that RC alleviates MI injury due to its multitarget and multicomponent activity. Therefore, in the present study, network pharmacology and experimental validation were performed to conduct systematic analysis of RC at the molecular level, to reveal the effect and underlying pharmacological mechanism of RC for treating MI.

Moreover, we obtained a PPI network by merging the active component target PPI of RC and the potential target PPI of MI (Figure 2C). Then, according to the following parameters, that is DC, BC, EC, LAC, NC, and CC, the topological characteristics of the PPI network were analyzed. A total of 126 core targets were found (Figure 2E; Supplementary Table S2). We also constructed a component-core target network and found that 60 bioactive components regulated 12 core targets, including AR, ESR1, CDK2, HSP90AA1, HSPA8, EGFR, AKT1, PARP1, GRB2, MDM2, HSP90AB1, and HSPA1A, which are closely involved in the apoptotic process (Figure 3; Supplementary Table S3). Apoptosis, which can be triggered by MI, is an energy-dependent programmed process responsible for cell removal (Zhang et al., 2022). Mitochondrial dysfunction is an important part of apoptosis caused by MI and is related to the impaired permeability of the mitochondrial outer membrane, causing the release of apoptosis-related proteins, such as apoptosis-inducing factor, Bcl-2 proteins, and cytochrome c (Ong and Gustafsson, 2012; Del Re et al., 2019). We then performed GO enrichment and KEGG enrichment analyses of the crucial targets. GO enrichment analysis can be performed to reveal the biological mechanisms of the core targets in disease. GO enrichment analysis was performed based on the following three terms: biological processes, cellular component, and molecular function terms (Ashburner et al., 2000). The results of GO enrichment analysis showed that the protective effects of RC on inhibiting MI are achieved through the simultaneous activation of multiple biological processes, cellular components, and molecular functions (Figure 4A). Next, we used the KEGG database to demonstrate the systematic functions and biological relevance of the potential targets (Chen et al., 2015). The KEGG pathway enrichment analysis of core targets indicated that RC may ameliorate MI-induced apoptosis by regulating multiple pathways, including pathways in cancer, transcriptional misregulation in cancer, viral carcinogenesis, the PI3K/Akt signaling pathway, alcoholism, ubiquitin-mediated proteolysis, etc. (Figure 4B). Among the pathways, the PI3K/Akt signaling pathway primarily regulates cellular survival and downstream targets, including endothelial nitric oxide synthase (eNOS), the mammalian target of rapamycin (mTOR), the Bcl-2 family, and glycogen synthase kinase 3 beta (GSK-3β) (Ghafouri-Fard et al., 2022). The activation of the PI3K/Akt pathway during MI leads to a smaller infarct and inhibition of apoptosis (Cheng et al., 2019; Meng et al., 2019). Therefore, the main pathway of RC against MI may be the classical PI3K/Akt signaling pathway. Moreover, the PI3K/Akt pathway was enriched with 15 core targets (Figure 5). To clarify the potential antiapoptotic mechanism of RC against MI, we then performed experimental validation in addition to network pharmacology analysis. Among the bioactive components of RC (Figure 3), berberine, coptisine, THP, palmatine, and quercetin were reported to be cardioprotective (Ruan et al., 2020; Wen et al., 2020; Zhu et al., 2020; Albadrani et al., 2021). In this study, we conducted molecular docking of the 5 components with AKT1, the core target that relates to the PI3K/AKT pathway. The results showed that THP had the lowest binding energy with AKT1 among the 5 components. Moreover, based on the evidence that THP improves cardiac function after MI/RI and modulates PI3K/Akt-mediated autophagy in an ischemia/reperfusion model (Han et al., 2012; Wen et al., 2020), we selected THP as the bioactive component for subsequent experimental study. The network pharmacology results indicate that RC might inhibit the pathogenesis of MI by activating the PI3K/Akt signaling pathway. Thus, molecular docking analysis, an effective and rapid approach for forecasting the binding affinity between the components of TCM and their targets in view of the spatial structure of ligands and receptors, was performed to further validate the potential mechanism of THP against MI (Gan et al., 2019). As shown in Figure 6, the molecular docking results indicate that THP has high affinity with Akt, suggesting that activation of the PI3K/Akt pathway via effective activation of Akt may be closely related to the mechanism of THP against MI. These results further confirm that THP may be the representative component of RC, and exhibits potent inhibitory activity against MI by regulating Akt and its various downstream signaling pathways.

Next, to determine the effect of THP on OGD-induced injury, we first performed a CCK8 assay to assess whether THP had cellular toxicity on H9c2 cells. The results showed that the viability of H9c2 cells was higher than 80% when H9c2 cells were treated with 3.75–240 μM THP. This result indicated that THP had no remarkable toxicity for H9c2 cells and could be used for further experiments. Further experiments showed that THP significantly reduced cardiomyocyte apoptosis after OGD exposure, as indicated by the recovery of cell viability (Figure 7), the decreased ratio of cleaved caspase-3 protein expression to caspase-3 protein expression, and reduced mRNA levels of Bax, Bcl-2 and p53 (Figure 8). IL-6, IL-1β, and TNFα are crucial cytokines in the inflammatory response and immune reaction of MI patients (Di Stefano et al., 2009; Silvain et al., 2020). Therefore, we investigated the expression of IL-6, IL-1β, and TNFα using RT-qPCR analysis and found that their expression was significantly augmented by the OGD stimulus and observably diminished by THP treatment in a dose-dependent manner (Figure 8). THP may exhibit anti-inflammatory and cardioprotective effects by downregulating the expression of IL-6, IL-1β, and TNFα. The PI3K/Akt pathway is closely associated with the oxidation and reduction of intracellular mitochondria and is a key regulatory pathway for protein synthesis *in vivo* (Chi et al., 2019). This pathway is inhibited in the myocardial cells of rats with MI, causing an apparent increase in myocardial apoptosis (Fujio et al., 2000; Wu et al., 2000; Yamashita et al., 2001). The results from KEGG enrichment showed that the PI3K/Akt signaling pathway may be closely related to the treatment of RC against OGD exposure, and most components had the potential to regulate Akt. However, THP regulation via the PI3k/Akt pathway needs to be comprehensively investigated. In this study, we found that THP treatment could lead to a higher phosphorylation level of Akt (Figure 8A). Further experiments demonstrated that blocking the PI3K/Akt pathway with the specific inhibitor LY294002 could diminish the anti-inflammatory and antiapoptotic effects of THP (Figures 8–10). These results indicated that THP protected against OGD-induced cardiac injury by increasing the phosphorylation level of Akt and decreasing its various downstream inflammatory factors, such as IL-6, IL-1β, and TNF-α.

However, there were some shortcomings in the study: 1) The internal absorption and utilization of RC is a complicated process, meaning the function of RC may not simply accumulate the effects of multiple compounds. 2) The predicted targets are greatly affected by the quality of the databases and the algorithm. 3) The anti-apoptotic effect and the exact mechanism of THP against MI should be further demonstrated with animal models and AKT knockout animals.

## Conclusion

In conclusion, the present study aimed to investigate the effect and mechanism of RC against MI via network pharmacology analysis, experimental validation and molecular docking studies. We identified 60 bioactive components with 431 potential targets and 1,131 MI-related targets through network pharmacology analysis. Then, 126 core targets were screened by PPI network topology analysis. The KEGG enrichment results and the experimental validation results suggested that RC achieves the protective effect against MI via the PI3K/Akt signaling pathway. The molecular mechanisms by which RC inhibits MI were associated with a high binding affinity between THP and AKT. In light of these findings, understanding the function and mechanism of RC can provide novel insight for therapeutic strategies to treat MI.

## Data Availability

The datasets presented in this study can be found in online repositories. The names of the repository/repositories and accession number(s) can be found in the article/Supplementary Material.

## Supplementary Material

Supplementary Figure S1 Component-target network. There were 60 bioactive components and 431 potential targets of RC. The orange nodes represent the potential active ingredients, and the violet nodes represent the core targets.

Supplementary Figure S2 Molecular docking diagram of Akt with berberine, coptisine, palmatine, and quercetin. Structure diagram of Akt docked **(A)** berberine, **(B)** coptisine, **(C)** palmatine, and **(D)** quercetin. Yellow lines represent hydrogen bond. **(E)** The binding energy for berberine, coptisine, palmatine, and quercetin docked into the Akt crystal structure.

## Glossary

Akt: protein kinase B
Bcl2: B cell lymphoma-2
Bax: BCL2 associated X
BC: betweenness centrality
CCK8: cell counting Kit-8
CC: closeness centrality
CABG: coronary artery bypass grafting
DC: degree centrality
DL: drug-likeness
EC: eigenvector centrality
ETCM: encyclopedia of traditional Chinese medicine
GEO: gene expression omnibus
GO: gene ontology
IL-1β: interleukin-1 beta
IL-6: interleukin-6
KEGG: Kyoto encyclopedia of genes and genomes
LAC: local average connectivity-based method centrality
MI: myocardial infarction
MI/RI: myocardial ischemia/reperfusion injury
NC: network centrality
OB: oral bioavailability
OGD: oxygen and glucose deprivation
PCI: percutaneous coronary intervention
PI3K: phosphatidylinositol 3-kinase
PPI: protein-protein interaction
RC: *Rhizoma corydalis*
TUNEL: TdT-mediated dUTP nick-end labeling
THP: tetrahydropalmatine
TTD: therapeutic target database
TCM: traditional Chinese medicine
TCMID: traditional Chinese medicine integrated database
TCMSP: traditional Chinese medicine systems pharmacology
TNF-α: tumor necrosis factor-α
