# Supplementary figures and images for: Uncovering the Effect and Mechanism of Rhizoma Corydalis on Myocardial Infarction Through an Integrated Network Pharmacology Approach and Experimental Verification
Source: Front Pharmacol. 2022 Jul 22;13:927488. doi: 10.3389/fphar.2022.927488 (PMC9355031; doi:10.3389/fphar.2022.927488)

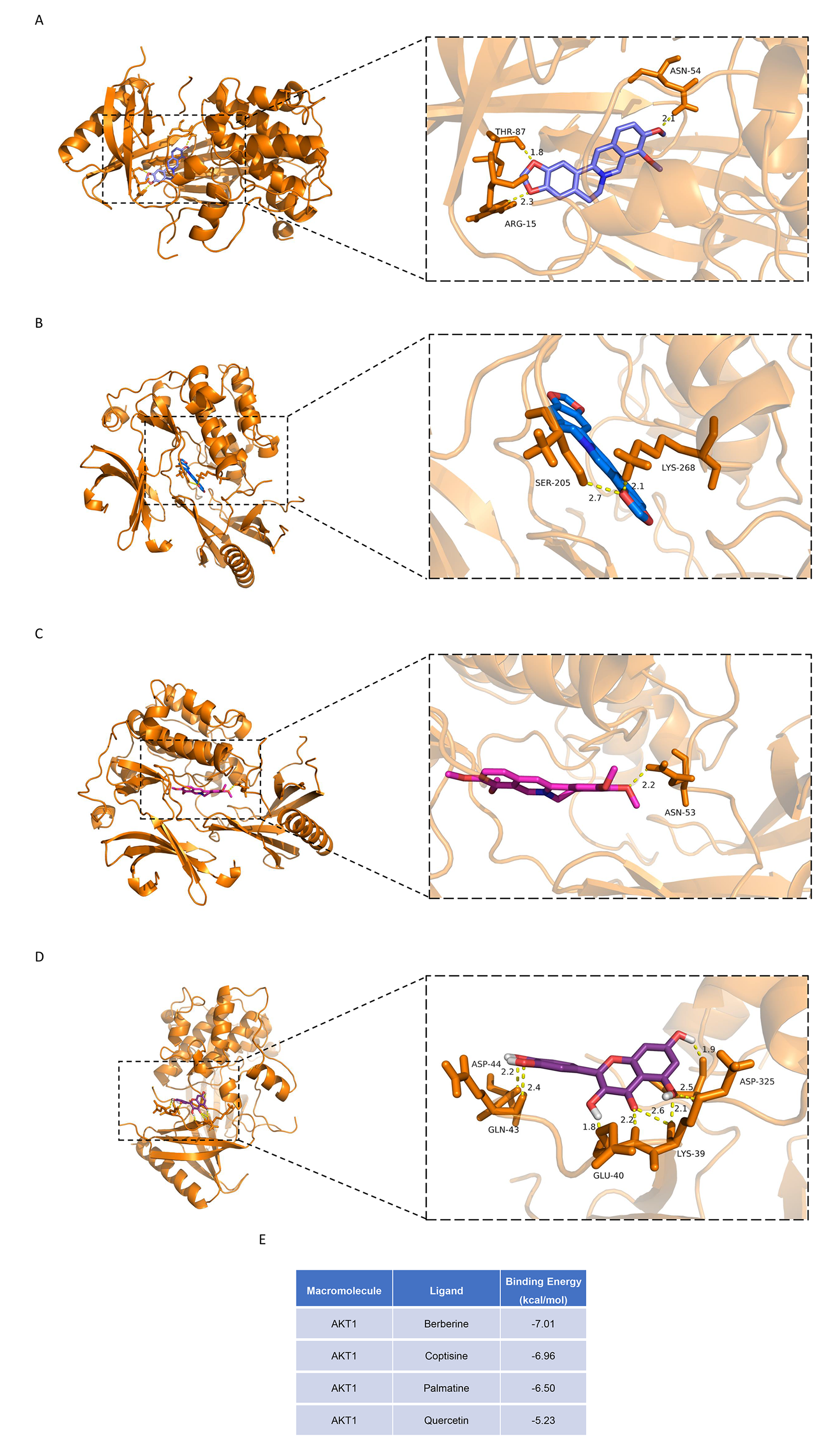

Supplement: Supplementary file 1 [file Image2.tif]

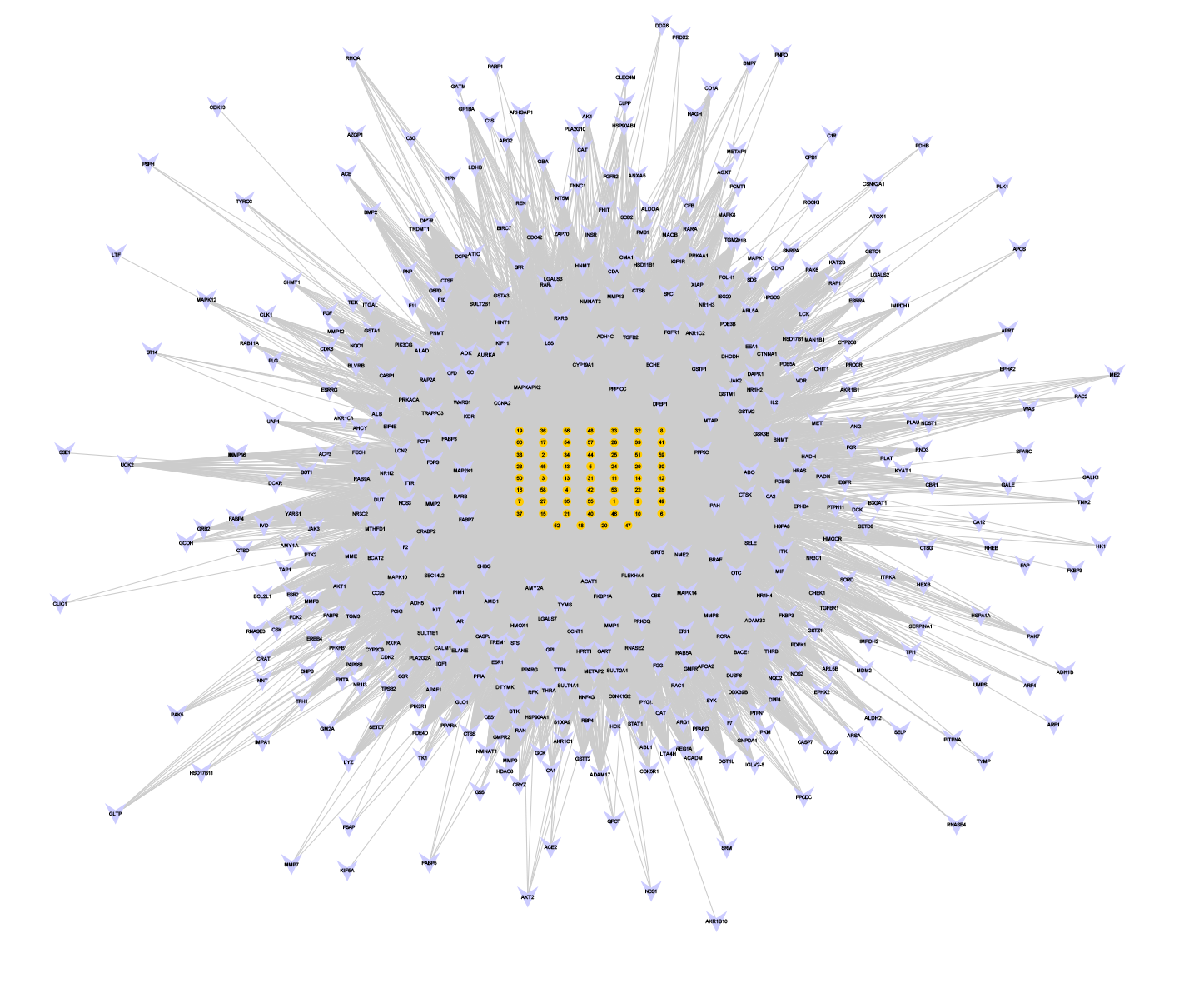

Supplement: Supplementary file 2 [file Image1.tif]
