# Supplementary material for: Uncovering the Effect and Mechanism of Rhizoma Corydalis on Myocardial Infarction Through an Integrated Network Pharmacology Approach and Experimental Verification
Source: Front Pharmacol. 2022 Jul 22;13:927488. doi: 10.3389/fphar.2022.927488 (PMC9355031; doi:10.3389/fphar.2022.927488)
Supplement: Supplementary file 3 [file DataSheet1.pdf]

Table S1. Bioactive ingredients of RC.

ID	Molecule Name	MW	Molecular Formula	pubchem CID
1	Berberine	336.39	$C_{20}H_{18}NO_4^+$	2353
2	Coptisine	320.34	$C_{19}H_{14}NO_4^+$	72322
3	Cryptopin	369.45	$C_{21}H_{23}NO_5$	72616
4	Dihydrochelerythrine	349.41	$C_{21}\ H_{19}\ NO_4$	485077
5	Dihydrosanguinarine	333.36	$C_{20}H_{15}NO_4$	124069
6	Sanguinarine	332.35	$C_{20}H_{14}NO_4^+$	5154
7	(S)-Scoulerine	327.41	$C_{19}H_{21}NO_4$	439654
8	Cavidine	353.45	$C_{21}H_{23}NO_4$	193148
9	(R)-Canadine	339.42	$C_{20}H_{21}NO_4$	443422
10	Sitosterol	414.79	$C_{29}H_{50}O$	12303645
11	Tetrahydropalmatine	355.47	$C_{21}H_{25}NO_4$	72301
12	Capaurine	371.47	$C_{21}H_{25}NO_5$	94149
13	Clarkeanidine	327.41	$C_{19}H_{21}NO_4$	127376
14	Corydaline	369.5	$C_{22}H_{27}NO_4$	101301

15	Corydalmine	340.45	$C_{20}H_{23}NO_4$	161665
16	Corydine	341.44	$C_{20}H_{23}NO_4$	10153
17	Corynoline	367.43	$C_{21}H_{21}NO_5$	177014
18	Corynoloxine	365.41	$C_{21}H_{19}NO_5$	1.01E+08
19	Methyl-[2-(3,4,6,7-tetramethoxy-1-phenanthryl)ethyl]amine	355.47	$C_{21}H_{25}NO_4$	11462401
20	Dehydrocavidine	351.43	$C_{21}H_{21}NO_4$	92043552
21	Dehydrocorybulbine	352.44	$C_{21}H_{22}NO_4^+$	5316439
22	Dehydrocorydaline	366.47	$C_{22}H_{24}NO_4^+$	34781
23	Dehydrocorydalmine	338.41	$C_{20}H_{20}NO_4^+$	3083983
24	13-Methyldehydrocorydalmine	352.44	$C_{21}H_{22}NO_4^+$	25254728
25	(1S,8'R)-6,7-dimethoxy-2-methylspiro[3,4-dihydroisoquinoline-1,7'-6,8-dihydrocyclopenta[g][1,3]benzodioxole]-8'-ol	369.45	$C_{21}H_{23}NO_5$	21770852
26	Isoboldine	327.41	$C_{19}H_{21}NO_4$	133323
27	13-Methylpalmatrubine	352.44	$C_{21}H_{22}NO_4^+$	12275616
28	N-methyllaurotetanine	341.44	$C_{20}H_{23}NO_4$	6543699
29	Norglaucin	341.44	$C_{20}H_{23}NO_4$	30835
30	Pontevedrine	381.41	$C_{21}H_{19}NO_6$	11047165

31	Pseudocoptisine	320.34	$C_{19}H_{14}NO_4^+$	15520811
32	Pseudoprotopine	353.4	$C_{20}H_{19}NO_5$	185559
33	Saulatine	396.47	$C_{22}H_{23}NO_6$	185141
34	l-Tetrahydrocoptisine	323.37	$C_{19}H_{17}NO_4$	697545
35	Tetrahydrocorysamine	337.4	$C_{20}H_{19}NO_4$	14315597
36	Tetrahydroprotopapaverine	329.43	$C_{19}H_{23}NO_4$	40512630
37	(+)-Thaliporphine	341.44	$C_{20}H_{23}NO_4$	6992288
38	2,3,9,10-Tetramethoxy-13-methyl-5,6-dihydroisoquinolino[2,1-b]isoquinolin-8-one	381.46	$C_{22}H_{23}NO_5$	10362429
39	Stigmasterol	412.77	$C_{29}H_{48}O$	5280794
40	Palmatine	352.44	$C_{21}H_{22}NO_4^+$	19009
41	Fumarine	353.4	$C_{20}H_{19}NO_5$	4970
42	Isocorypalmine	341.44	$C_{20}H_{23}NO_4$	440229
43	Bicuculline	367.38	$C_{20}H_{17}NO_6$	10237
44	Bulbocapnine	325.39	$C_{19}H_{19}NO_4$	12441
45	Quercetin	302.25	$C_{15}H_{10}O_7$	5280343
46	Fumaric Acid	116.072 2	$C_4H_4O_4$	444972

47	Allocryptopine	369.411	$C_{21}H_{23}NO_5$	98570
48	Columbamine	338.3771	$C_{20}H_{20}NO_4^+$	72310
49	(S)-Canadine	339.385	$C_{20}H_{21}NO_4$	21171
50	Corybulbine	355.4275	$C_{21}H_{25}NO_4$	10316181
51	Corypalmine	341.4009	$C_{20}H_{23}NO_4$	11186895
52	Glaucine	355.4275	$C_{21}H_{25}NO_4$	16754
53	Isocorydine	341.4009	$C_{20}H_{23}NO_4$	10143
54	(+)-Isocorypalmine	341.4009	$C_{20}H_{23}NO_4$	71261649
55	Leonticine	327.4174	$C_{20}H_{25}NO_3$	12314123
56	N-Methyllaurotetanine	341.400	$C_{20}H_{23}NO_4$	16573

		9		
57	Norisocorydine	327.374<br>3	$C_{19}H_{21}NO_4$	12313549
58	(S)-Stylopine	323.342<br>6	$C_{19}H_{17}NO_4$	440583
59	Yuanhunine	355.427<br>5	$C_{21}H_{25}NO_4$	128558
60	d-Corydalin	369.5	$C_{22}H_{27}NO_4$	326549

Table S2. Information of 126 core targets.

Gene name	Description
CDK2	cyclin dependent kinase 2
CUL5	cullin 5
RPA1	replication protein A1
ILF2	interleukin enhancer binding factor 2
RPA3	replication protein A3
CAND1	cullin associated and neddylation dissociated 1
RPA2	replication protein A2
SYNCRIP	synaptotagmin binding cytoplasmic RNA interacting protein
ILF3	interleukin enhancer binding factor 3
NEDD8	neural precursor cell expressed, developmentally down-regulated 8
RPS3	ribosomal protein S3
RPS6	ribosomal protein S6
COPS5	COP9 signalosome subunit 5
RPS4X	ribosomal protein S4, X-linked
MYH9	myosin heavy chain 9
TARDBP	TAR DNA binding protein
HSPA1A	heat shock protein family A (Hsp70) member 1A
SF3B1	splicing factor 3b subunit 1
HSPA1B	heat shock protein family A (Hsp70) member 1B
KAT5	lysine acetyltransferase 5
MYC	v-myc avian myelocytomatosis viral oncogene homolog
PRKDC	protein kinase, DNA-activated, catalytic polypeptide
CEP250	centrosomal protein 250
HSP90AA1	heat shock protein 90 alpha family class A member 1
HSP90AB1	heat shock protein 90 alpha family class B member 1
HSPA5	heat shock protein family A (Hsp70) member 5
CLTC	clathrin heavy chain

HSPA4	heat shock protein family A (Hsp70) member 4
HSPA9	heat shock protein family A (Hsp70) member 9
RPS27A	ribosomal protein S27a
HSPA8	heat shock protein family A (Hsp70) member 8
PARP1	poly(ADP-ribose) polymerase 1
NPM1	nucleophosmin
FLNA	filamin A
UBL4A	ubiquitin like 4A
HIST1H4I	histone cluster 1 H4 family member i
HNRNPM	heterogeneous nuclear ribonucleoprotein M
NTRK1	neurotrophic receptor tyrosine kinase 1
EED	embryonic ectoderm development
NCL	nucleolin
FN1	fibronectin 1
TUBG1	tubulin gamma 1
ACTB	actin beta
H2AFX	H2A histone family member X
HUWE1	HECT, UBA and WWE domain containing 1, E3 ubiquitin protein ligase
UBE2I	ubiquitin conjugating enzyme E2 I
EGFR	epidermal growth factor receptor
FBXO6	F-box protein 6
MAP3K1	mitogen-activated protein kinase kinase kinase 1
AKT1	AKT serine/threonine kinase 1
GRB2	growth factor receptor bound protein 2
MDM2	MDM2 proto-oncogene
CUL7	cullin 7
HDAC1	histone deacetylase 1
MCM5	minichromosome maintenance complex component 5

MCM2	minichromosome maintenance complex component 2
HDAC2	histone deacetylase 2
AR	androgen receptor
CCDC8	coiled-coil domain containing 8
CREBBP	CREB binding protein
VCP	valosin containing protein
STAU1	staufen double-stranded RNA binding protein 1
VHL	von Hippel-Lindau tumor suppressor
SNW1	SNW domain containing 1
EP300	E1A binding protein p300
SIRT7	sirtuin 7
VCAM1	vascular cell adhesion molecule 1
HIST1H3F	histone cluster 1 H3 family member f
ARRB2	arrestin beta 2
UBC	ubiquitin C
ITGA4	integrin subunit alpha 4
PABPC1	poly(A) binding protein cytoplasmic 1
XPO1	exportin 1
XRCC5	X-ray repair cross complementing 5
YWHAG	tyrosine 3-monooxygenase/tryptophan 5-monooxygenase activation protein gamma
YWHAE	tyrosine 3-monooxygenase/tryptophan 5-monooxygenase activation protein epsilon
PAN2	PAN2 poly(A) specific ribonuclease subunit
SUZ12	SUZ12 polycomb repressive complex 2 subunit
U2AF2	U2 small nuclear RNA auxiliary factor 2
EEF1A1	eukaryotic translation elongation factor 1 alpha 1
PARK2	parkin RBR E3 ubiquitin protein ligase
HNRNPU	heterogeneous nuclear ribonucleoprotein U

SRRM2	serine/arginine repetitive matrix 2
HIST1H4H	histone cluster 1 H4 family member h
CTNNB1	catenin beta 1
HNRNPK	heterogeneous nuclear ribonucleoprotein K
ESR1	estrogen receptor 1
HNRNPA1	heterogeneous nuclear ribonucleoprotein A1
YWHAZ	tyrosine 3-monooxygenase/tryptophan 5-monooxygenase activation protein zeta
SMURF1	SMAD specific E3 ubiquitin protein ligase 1
HDAC5	histone deacetylase 5
EZH2	enhancer of zeste 2 polycomb repressive complex 2 subunit
APP	amyloid beta precursor protein
EWSR1	EWS RNA binding protein 1
RELA	RELA proto-oncogene, NF-kB subunit
FUS	FUS RNA binding protein
CUL4B	cullin 4B
CUL4A	cullin 4A
CUL3	cullin 3
CUL2	cullin 2
TUBB	tubulin beta class I
CUL1	cullin 1
HIST1H3J	histone cluster 1 H3 family member j
HIST1H3E	histone cluster 1 H3 family member e
HIST1H3B	histone cluster 1 H3 family member b
XRCC6	X-ray repair cross complementing 6
HIST1H3H	histone cluster 1 H3 family member h
TP53	tumor protein p53
HIST1H3A	histone cluster 1 H3 family member a
HIST1H3D	histone cluster 1 H3 family member d

RACK1	receptor for activated C kinase 1
EIF4A3	eukaryotic translation initiation factor 4A3
HIST1H3G	histone cluster 1 H3 family member g
BMI1	BMI1 proto-oncogene, polycomb ring finger
HIST1H3C	histone cluster 1 H3 family member c
SMARCA4	SWI/SNF related, matrix associated, actin dependent regulator of chromatin, subfamily a, member 4
HIST1H3I	histone cluster 1 H3 family member i
BRCA1	BRCA1, DNA repair associated
RNF2	ring finger protein 2
CDC5L	cell division cycle 5 like
OBSL1	obscurin like 1
YWHAQ	tyrosine 3-monooxygenase/tryptophan 5-monooxygenase activation protein theta
IKBKG	inhibitor of nuclear factor kappa B kinase subunit gamma
DDX5	DEAD-box helicase 5
TRAF6	TNF receptor associated factor 6
DHX9	DExH-box helicase 9

Table S3. Core targets and corresponding bioactive components.

Gene Name	Number of Component	Components ID
HSP90AA1	60	1, 2, 3, 4, 5, 6, 7, 8, 9, 10, 11, 12, 13, 14, 15, 16, 17, 18, 19, 20, 21, 22, 23, 24, 25, 26, 27, 28, 29, 30, 31, 32, 33, 34, 35, 36, 37, 38, 39, 40, 41, 42, 43, 44, 45, 46, 47, 48, 49, 50, 51, 52, 53, 54, 55, 56, 57, 58, 59, 60
AR	59	1, 2, 3, 4, 5, 6, 7, 8, 9, 10, 11, 12, 13, 14, 15, 16, 17, 18, 19, 20, 21, 22, 23, 24, 25, 26, 27, 28, 29, 30, 31, 32, 33, 34, 35, 36, 37, 38, 39, 40, 41, 42, 43, 44, 45, 47, 48, 49, 50, 51, 52, 53, 54, 55, 56, 57, 58, 59, 60
CDK2	59	1, 2, 3, 5, 6, 7, 8, 9, 10, 11, 12, 13, 14, 15, 16, 17, 18, 19, 20, 21, 22, 23, 24, 25, 26, 27, 28, 29, 30, 31, 32, 33, 34, 35, 36, 37, 38, 39, 40, 41, 42, 43, 44, 45, 46, 47, 48, 49, 50, 51, 52, 53, 54, 55, 56, 57, 58, 59, 60
ESR1	58	1, 2, 3, 4, 5, 6, 7, 8, 9, 10, 11, 12, 13, 14, 15, 16, 17, 18, 19, 20, 21, 22, 23, 24, 25, 26, 27, 28, 29, 31, 32, 33, 34, 35, 36, 37, 38, 39, 40, 41, 42, 43, 44, 45, 47, 48, 49, 50, 51, 52, 53, 54, 55, 56, 57, 58, 59, 60
EGFR	55	1, 2, 4, 5, 6, 7, 8, 9, 10, 11, 12, 13, 14, 15, 16, 17, 18, 19, 20, 21, 22, 23, 24, 25, 26, 27, 28, 29, 30, 31, 33, 34, 35, 36, 37, 38, 39, 40, 42, 43, 44, 45, 48, 49, 50, 51, 52, 53, 54, 55, 56, 57, 58, 59, 60
HSPA8	39	1, 3, 4, 8, 9, 10, 12, 14, 15, 16, 17, 20, 21, 22, 23, 24, 25, 27, 31, 32, 33, 34, 35, 36, 38, 40, 41, 43, 44, 45, 46, 47, 48, 50, 53, 54, 57, 59, 60
AKT1	35	3, 4, 7, 12, 13, 15, 16, 18, 19, 21, 23, 24, 25, 26, 27, 28, 29, 30, 32, 33, 36, 37, 38, 40, 41, 44, 45, 46, 47,

		50, 52, 53, 55, 56, 57, 59
GRB2	22	7, 8, 10, 14, 15, 17, 18, 19, 20, 21, 22, 24, 27, 35, 38, 39, 42, 48, 50, 54, 59, 60
HSP90AB1	22	8, 10, 14, 15, 17, 18, 20, 21, 22, 23, 24, 26, 27, 28, 35, 38, 39, 48, 50, 56, 59, 60
MDM2	17	8, 10, 14, 17, 18, 20, 21, 22, 24, 27, 33, 35, 38, 39, 50, 59, 60
HSPA1A	3	36,38,46
PARP1	2	4,10
